# Er-Miao-Fang Extracts Inhibits Adipose Lipolysis and Reduces Hepatic Gluconeogenesis via Suppression of Inflammation

**DOI:** 10.3389/fphys.2018.01041

**Published:** 2018-08-14

**Authors:** Wenjun Zhao, Xin Feng, Baolin Liu, Jiechen Xian, Ning Zhang

**Affiliations:** ^1^Experiment Center for Science and Technology, Shanghai University of Traditional Chinese Medicine, Shanghai, China; ^2^Shanghai Key Laboratory of Regulatory Biology, Institute of Biomedical Sciences and School of Life Sciences, East China Normal University, Shanghai, China; ^3^Clinical Metabolomics Centre, China Pharmaceutical University, Nanjing, China; ^4^Engineering Research Center of Modern Preparation Technology of Traditional Chinese Medicine, Shanghai University of Traditional Chinese Medicine, Shanghai, China

**Keywords:** Er-Miao-Fang, inflammation, PDE3B, PDE4B, glucagon, gluconeogenesis

## Abstract

High-fat-diet (HFD) feeding induces adipose dysfunction. This study aims to explore whether the Traditional Chinese Medical prescription Er-Miao-Fang could ameliorate adipose dysfunction and prevent hepatic glucose output. Short-term HFD feeding induced adipose lipolysis accompanied with enhanced hepatic glucose output in mice. Adipose lipolysis is initiated by cyclic adenosine monophosphate (cAMP)/protein kinase A (PKA) signaling. Oral administration Er-Miao-Fang inhibited inflammation in adipose tissue by dephosphorylation of JNK and reducing TNF-α and IL-1β production, and thus preserved phosphodiesterase 3B (PDE3B) induction, contributing to preventing cAMP accumulation. As a result, from suppression of PKA activation, Er-Miao-Fang reduced fatty acids and glycerol release from adipose tissue due to the inhibition hormone-sensitive lipase (HSL). By blocking the traffic of fatty acids and inflammatory mediators from adipose tissue to the liver, Er-Miao-Fang attenuated hepatic cAMP/PKA signaling by protecting phosphodiesterase 4B (PDE4B) induction from inflammatory insult, and thereby reduced hepatic glucose production by suppression of hepatic glucagon response in HFD-fed mice. In conclusion, Er-Miao-Fang prevented adipose lipolysis by suppression of inflammation, contributing to reducing excessive hepatic glucose output. These findings present a new view of regulating gluconeogenesis and provide the guiding significance for the regulation of multi-link targets with Traditional Chinese Medicine.

## Introduction

Adipose tissue functions as a site of fat storage, while adipose dysfunction in obesity and diabetes induces lipolysis and increases circulating free fatty acids to promote ectopic fat deposits. It is generally accepted that lipid accumulation in the liver and muscle is the main cause for insulin resistance (Lafontan and Girard, 2008; Kowalski et al., 2015; Zhao et al., 2016). The action of insulin in liver is to suppress hepatic glucose production and lipolysis-induced lipid accumulation in the liver is shown to attenuate insulin sensitivity and increase hepatic glucose production, responsible for hyperglycemia during fasting in diabetes (Perry et al., 2014). These events suggest the functional interaction between adipose lipolysis and abnormal hepatic gluconeogenesis.

Several hormones and effectors can induce lipolysis in adipose tissue though the activation of cAMP-dependent protein kinase A (PKA), in which hormone-sensitive lipase (HSL), a key enzyme in the mobilization of fatty acids from stored triacylglyceride (TG), is activated (Greenberg et al., 2001; Greenberg et al., 2011; Schweiger et al., 2014). Insulin regulates post-prandial glucose levels by promoting glucose disposal, while maintains glucose homeostasis during fasting conditions by promoting hepatic glucose output. Several lines of evidence demonstrate that enhanced hepatic glucagon response is responsible for hyperglycemia during fasting (Unger and Cherrington, 2012). In liver, hepatic gluconeogenesis responded to glucagon is also initiated by cAMP/PKA signaling, transcriptionally regulating gene encoding to gluconeogenesis enzymes, including G6Pase and PEPCK (Leahy et al., 1999; Streeper et al., 2001; Yang and Yang, 2016). Blocking cAMP/PKA signaling suppresses HSL activation to inhibit lipolysis (Zhao et al., 2016). Hepatic glucagon response is restrained by blocking cAMP/PKA signaling and inhibiting transcriptional regulation of G6Pase and PEPCK (He et al., 2009). All these well elucidate that cAMP/PKA signaling plays a vital role in regulation of lipid and glucose metabolism.

As a second messenger, cAMP is synthesized by adenylate cyclase (AC) while phosphodiesterases (PDEs) could prevent cAMP accumulation through degradation. PDEs are invariably diverse, for instance, PDE3 and PDE4 provide the major portion of cAMP hydrolyzing activity in most cells (Francis et al., 2011). PDE3B is proposed to be the predominant isoform of PDEs in adipose tissue. Inflammation is demonstrated to regulate PDE3B induction. TNF-α inhibited PDE3B activity with suppression of PDE3B induction and thus increases lipolysis, indicative of the involvement of inflammation in lipolysis (Mei et al., 2002). In the liver, the members of the PDE3 and PDE4 subfamilies are both expressed. While PDE4B is the predominant regulator for hepatic cAMP degradation (Miller et al., 2013). Similarly, it is documented that inhibiting cAMP accumulation by preserving PDE4B activity contributes to suppress hepatic glucagon response (Xiao et al., 2017). So, decreased PDEs expression is associated with the dysfunction in adipose and liver, and these events emphasize the possible relevance of inflammation in the regulation of fatty acid and hepatic glucagon response.

Er-Miao-Fang is a famous Traditional Chinese Medical prescription first recorded in Dan Xi Xin Fa in 1481. It is composed of two medicinal herbs: *Phellodendron chinense* Schneid and *Atractylodes lancea* (Thunb.) DC or *Atractylodes chinensis* (DC.) Koidz and it has been widely used to treat arthritis, urinary tract infections, and diarrhea for decades. Studies about the pharmacological mechanism of this ancient prescription are performed mainly focused on its anti-inflammatory activity (Chen et al., 2014; Bae et al., 2015). The alkaloids are the main components in the Er-Miao-Fang extracts with abundant berberine (4.019%) and phellodendrine (0.371%), the main effective ingredients (Feng et al., 2017). Berberine was documented to reduce lipid droplet accumulation and improve insulin sensitivity (Lee et al., 2006). Treatment of berberine reduces the levels of fasting blood glucose and inhibits the expression of G6Pase and PEPCK (Wei et al., 2016). Phellodendrine ameliorated oxidative stress by downregulating NF-κB phosphorylation (Li et al., 2016). These studies suggest the potential role of Er-Miao-Fang extracts in the metabolism disorder. Hence, in this study, we explored the possible pharmacological action of Er-Miao-Fang extracts in regulation of lipolysis and hepatic gluconeogenesis by inhibiting adipose inflammation.

## Materials and Methods

### Preparation of Er-Miao-Fang

*Phellodendron chinense* Schneid and *A. lancea* (Thunb.) DC. were purchased from Shanghai Kangqiao Chinese Medicine Yinpian Co., Ltd. (Shanghai, China) and authenticated by Yan Ke (Experiment Center for Teaching and Learning, Shanghai University of Traditional Chinese Medicine). Qualities of the crude drugs meet the standards of “Pharmacopoeia of the People’s Republic of China” (2015 edition). *P. chinense* Schneid with the same weight of *A. lancea* (Thunb.) DC. was boiled three times with 10 times the volume (*v/w*) of 70% ethanol for 2 h each time after soaking 30 min. The extracts were filtered, concentrated and decompression vacuum drying fewer than 65°C was adopted. Er-Miao-Fang extracts were prepared and the yield was 24.7%. The chemical components of Er-Miao-Fang extracts were analyzed by UPLC-MS/MS, and the followings are the chief six components and contents tested in the extracts: berberine, 4.019%; phellodendrine, 0.371%; chlorogenic acid, 0.158%; ferulic acid, 0.141%; magnoflorine, 0.107 %; and palmatine, 0.039% (Feng et al., 2017).

### Reagents

Metformin (purity ≥ 99%) was purchased from Sangon Biotech (Shanghai, China) and dissolved in 0.3% (*w/v*) sodium carboxymethylcellulose (CMC-Na) for animal administration or in dimethyl sulfoxide (DMSO) for cell experiment [the final concentration of DMSO was 0.1% (*v/v*)]. Glucagon (purity ≥ 98%) was obtained from Kinase Chemicals Ltd. (Suffolk, United Kingdom). Sodium pyruvate (purity ≥ 99%) was provided by Sigma-Aldrich (Shanghai, China). Palmitate (PA, Sinopharm, Shanghai, China) was dissolved in ethanol to prepare 200 mM stock solution, and then diluted with medium containing 10% non-esterified fatty acid (NEFA)-free bovine serum albumin (BSA) before use (*v/v*, 1:19). Mouse cAMP, AMP, TNF-α, and IL-1β ELISA kits were provided by Shuojia Biotechnology Co., Ltd. (Shanghai, China). The following items were purchased from the cited commercial sources: anti-phospho-(Ser/Thr) PKA substrate (9621), anti-phospho-HSL (Ser660) (4126), anti-HSL (4107), anti-phospho-SAPK/JNK (Thr183/Tyr185) (4668), anti-SAPK/JNK (9252), anti-β-actin (4970), Cell Signaling Technology (Beverly, MA, United States); anti-TNF-α (ab6671), anti-CREB (phospho S133) (ab32096), anti-CREB (ab32515), goat anti-rabbit IgG H&L (Alexa Fluor^®^488) (ab150077), Abcam (Cambridge, MA, United States); PDE3B (H-300) (sc-20793), PDE4B (H-56) (sc-25812), Santa Cruz Biotechnology (Dallas, TX, United States); peroxidase-conjugated affinipure goat anti-Rabbit IgG (H&L) (111-035-003), Jackson ImmunoResearch Laboratories Inc. (West Grove, PA, United States); mouse IL-1β (AF-401-NA), R&D System (Minneapolis, MN, United States); RNAiso Plus (9108), RT Reagent Kit (RR037A), SYBR^®^ Premix Ex Taq (RR420A), Takara Bio Inc. (Dalian, China).

### Animals

Male ICR mice (6 weeks) were purchased from Sino-British Sippr/BK Lab. Animal Ltd. (Shanghai, China, production license: SCXK (Shanghai) 2013-0016). Mice were housed with 12 h light/dark cycles under a constant temperature (22 ± 2°C) and free access to water and food. This study was carried out in accordance with the recommendations of Provision and General Recommendation of Chinese Experimental Animals Administration Legislation. The protocol was approved by Animal Ethics Committee of Shanghai University of Traditional Chinese Medicine.

Mice were fed a regular chow diet or high-fat-diet (HFD) (10% yolk, 10% Lard, 0.2% cholate, 1% cholesterol and 78.8% standard diet, 36.45% Kcal fat) for 10 days (Wang et al., 2016; Zhao et al., 2016) with oral administration of saline, Er-Miao-Fang extracts (1 g/kg) or metformin (200 mg/kg), respectively every day. Blood was collected after 8 h fasting and blood glucose was assayed with commercial kits (Jiancheng, Nanjing, China). Levels of insulin and glucagon in blood were tested using ELISA kits (Shuojia, Shanghai, China). Mice were sacrificed after fasting for 8 h and the epididymis adipose tissue and liver tissue were isolated or cultured for assay. Epididymal adipose tissue or liver tissue was grinded in RIPA lysis buffer (Beyotime, Haimen, China). The lysates were centrifuged for collection of supernatants. The contents of cAMP, AMP, TNF-α, and IL-1β in the supernatant were measured by commercial ELISA Kits. The results were normalized by the amount of protein.

### Preparation of Conditioned Medium (CM) of Adipose Tissue From HFD-fed Mice

Epididymis adipose tissue was collected from chow-fed or HFD-fed mice and cut into small pieces, then incubated with the same weight in 2 mL DMEM (25 mM glucose, Gibco, Grand Island, NY, United States) containing 10% FBS, 100 U/mL penicillin and 100 μg/mL streptomycin) for 24 h at 37°C in a 5% CO_2_ incubator (Wang et al., 2016; Zhao et al., 2016). Collected the DMEM, centrifuged at 3,000 *g* for 5 min at 4°C and the supernatant was harvested as conditioned medium (CM). For the preparation of glucose-free CM, the chow-fed or HFD-fed mice adipose tissue was incubated in Krebs-Ringer phosphate-HEPES buffer (KRH buffer, containing 118 mM NaCl, 5 mM KCl, 1.3 mM CaCl_2_, 1.2 mM MgSO_4_, 1.2 mM KH_2_PO_4_, and 30 mM HEPES, containing 0.5% BSA, pH 7.4). The CM was used to culture hepatocytes to explore the crosstalk between adipose and liver. The levels of free fatty acids and glycerol in CM were detected with commercial kits following the manufacturer’s instructions (Jiancheng, Nanjing, China).

### Isolated Adipose Tissue Treatment

To study the pathway whereby Er-Miao-Fang inhibits lipolysis *in vitro*, epididymis adipose tissue was collected from sacrificed normal male mice, cut into small pieces, and then incubated in DMEM. Isolated adipose tissue was treated with Er-Miao-Fang (100 μg/mL), TNF-α antibody (1 μg/mL), or IL-1β antibody (0.6 μg/mL) for 30 min before challenged with PA (100 μM) for 24 h. The adipose tissue was homogenized in PBS and cAMP contents in the suspension were measured by ELISA kits. The PDE3B in the isolated adipose tissue was extracted with RIPA lysis buffer (Beyotime, Haimen, China). The lysates were centrifuged and the expression of PDE3B in the supernatant was determined by Western blot.

### Glucose and Pyruvate Tolerance Tests

Oral glucose tolerance testing (GTT) was performed in mice using glucose (2 g/kg) after overnight fasting. For pyruvate tolerance test (PTT), mice were injected intraperitoneally with pyruvate (2 g/kg) after fasting for 16 h. Blood was collected at regular intervals for the assay of glucose contents and calculated blood glucose area under the curve (AUC-G) with the methods mentioned before (Zhao et al., 2014).

### Hepatocytes Culture and Measurement of Cellular cAMP

BNL CL.2 hepatocytes (Cell storeroom of Chinese Academy of Sciences, Shanghai, China) were cultured in DMEM and maintained at 37°C in a 5% CO_2_ incubator. The cells were treated with adipose-derived CM or TNF-α antibody (1 μg/mL) and IL-1β antibody (0.6 μg/mL) in the presence or absent of glucagon (100 nM) for 24 h. After treatment, cells were collected and extracted with RIPA lysis buffer. The lysates were centrifuged and the contents of cAMP in the supernatant were measured by commercial ELISA Kits.

### Hepatocytes Glucose Output

BNL CL.2 hepatocytes were cultured with glucose-free adipose-derived CM or TNF-α antibody (1 μg/mL) and IL-1β antibody (0.6 μg/mL) for 24 h. After washing, the cells were incubated in KRH buffer supplemented with 20 mM pyruvate, with or without 100 nM glucagon for 6 h. The supernatant was collected for glucose analysis with commercial kits.

### Quantitative Real Time RT-PCR

Total mRNA was extracted from tissue or cells using RNAiso plus following the manufacture’s protocol and cDNA synthesis were preformed using RT reagent kit. Relative cDNA levels were determined using the SYBR Premix Ex Taq and amplified with ABI 7500 system. Target mRNA was normalized by ribosomal 18s RNA, an endogenous control. PCR primers were used as follows: mouse *PGC-1α* (134 bp), Forward Primer: 5′-TATGGAGTGACATAGAGTGTGCT-3′, Reverse Primer: 5′-CCACTTCAATCCACCCAGAAAG-3′; mouse *Pepck* (159 bp), Forward Primer: 5′-CTGCATAACGGTCTGGACTTC-3′, Reverse Primer: 5′-CAGCAACTGCCCGTACTCC-3′; mouse G6pase (*G6pc*, 173 bp), Forward Primer: 5′-CGACTCGCTATCTCCAAGTGA-3′, Reverse Primer: 5′-GTTGAACCAGTCTCCGACCA-3′; mouse 18s RNA (151 bp), Forward Primer: 5′-GTAACCCGTTGAACCCCATT-3′, Reverse Primer: 5′-CCATCCAATCGGTAGTAGCG-3′. Relative quantification was calculated based on the following equation: relative quantification = 2ξ^-ΔΔC_t_^. Ct is the threshold cycle to detect fluorescence.

### Western Blot Analysis

Tissue or cells were homogenized in RIPA lysis buffer with PMSF (RIPA: PMSF = 100:1, v/v). The lysates were centrifuged for collection of supernatants. Bicinchoninic acid (BCA) Protein Assay Kit (Beyotime, Haimen, China) was used to test the supernatant proteins contents. The protein samples were separated by 10% SDS–PAGE, transferred to PVDF membranes (Millipore Co., Ltd. MA, United States), blocked with 5% BSA/TBST buffer (5 mM Tris-base, pH 7.6, 136 mM NaCl, 0.05% Tween-20), immunoblotted with primary and secondary antibody. ECL Western Blotting Detection System and Image-Pro Plus 6.0 software (IPP 6.0, IPWIN Applicaton, Inc., Rrockville, MD, United States) was applied to analysis antibody-antigen complexes. The original images are provided as **Supplementary Image 1**.

### Statistical Analysis

All the results were expressed as mean ± SD and were subjected to one-way ANOWA analysis of variance followed by Student-Newman-Keuls multiple comparison test if significant (IBM SPSS Statistics 21.0, SPSS Inc., Chicago, IL, United States). *p* < 0.05 was considered statistically significant.

## Results

### Er-Miao-Fang Attenuated Lipolysis in Adipose Tissue

**FIGURE 1:**
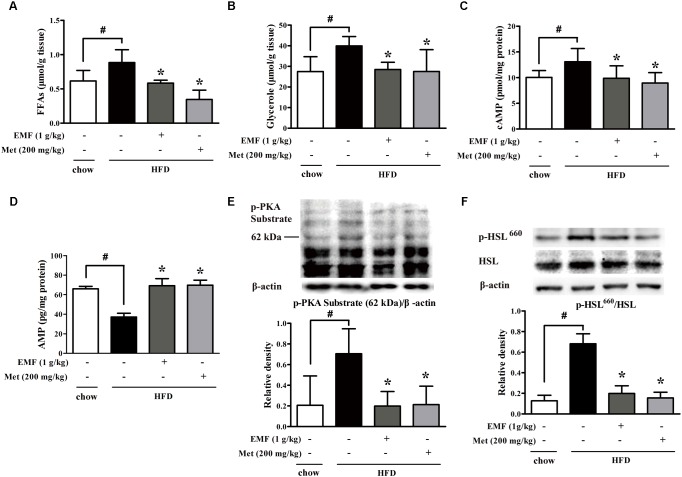
Er-Miao-Fang attenuated lipolysis in adipose tissue. Mice were fed with chow diet or HFD for 10 days with oral administration of Er-Miao-Fang extracts (EMF, 1 g/kg) or metformin (Met, 200 mg/kg). **(A,B)** Adipose tissue from chow diet fed or HFD fed mice were incubated in DMEM for 24 h. The fat-derived CM in DMEM was collected and detected the contents of free fatty acids (FFAs) and glycerol released from adipose tissue. The results were expressed as the mean ± SD (*n* = 5∼6). **(C,D)** cAMP and AMP in epididymal adipose tissue were determined by ELISA kits (mean ± SD, *n* = 5∼6). **(E)** Phosphorylation of PKA substrate and **(F)** Ser-660 motif of HSL in epididymal adipose tissue were detected by Western blot. The results were showed as the mean ± SD from four independent experiments. ^∗^*p* < 0.05 vs. HFD feeding only treatment, #*p* < 0.05 vs. the indicated treatment.

Short-term HFD feeding induced lipolysis from adipose tissue in mice, demonstrated by increased free fatty acids and glycerol released from isolated epididymal adipose tissue, whereas the increased lipolysis was inhibited by oral administration of Er-Miao-Fang extracts during HFD feeding (**Figures 1A,B**). Because adipose lipolysis is mediated by cAMP/PKA signaling, we examined the effects of Er-Miao-Fang in adipose tissue, and found that HFD feeding increased cAMP accumulation with reducing AMP contents in adipose tissue, whereas the changes were reversed by oral administration of Er-Miao-Fang (**Figures 1C,D**). HFD feeding induced PKA activation, but Er-Miao-Fang treatment inactivated PKA by dephosphorylation of PKA 62KDa substrate (**Figure 1E**). These results suggested that Er-Miao-Fang prevented PKA activation by downregulation of cAMP. The phosphorylation of PKA substrate at 62 KDa corresponds to the molecular weight of perilipin. As the major substrate for phosphorylation of PKA, perilipin facilitates lipolysis by HSL hydrolyzing triacylglycerol and diacylglycerol to induce fatty acid release (Miyoshi et al., 2006; Brasaemle, 2007; Gauthier et al., 2008). We found that oral administration of Er-Miao-Fang suppressed HSL activation by dephosphorylation (**Figure 1F**). These results suggested that Er-Miao-Fang prevented adipose lipolysis by blocking cAMP/PKA signaling. Anti-diabetic agent metformin also effectively reduced lipolysis from adipose tissue in HFD-fed mice.

### Er-Miao-Fang Inhibited Inflammation in Adipose Tissue

**FIGURE 2:**
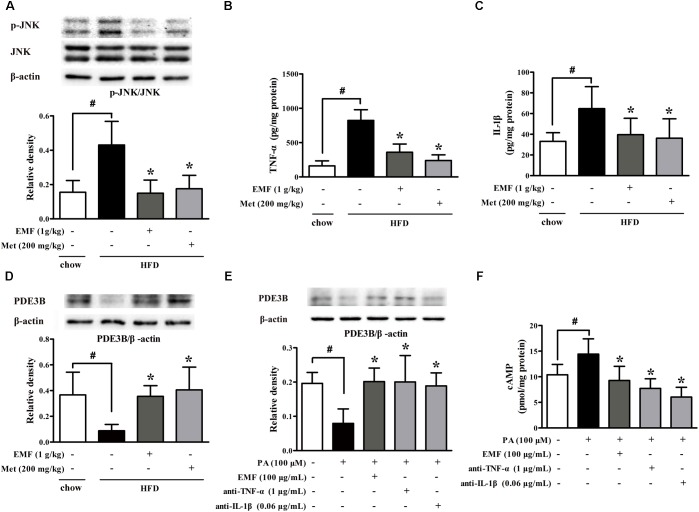
Er-Miao-Fang inhibited inflammation in adipose tissue. Mice were fed with chow diet or HFD for 10 days with oral administration of Er-Miao-Fang extracts (EMF, 1 g/kg) or metformin (Met, 200 mg/kg). **(A)** JNK protein expression was assessed by Western blot (*n* = 4). **(B,C)** TNF-α (*n* = 6) and IL-1β (*n* = 8∼10) were measured by ELISA kits. **(D)** PDE3B protein expression was determined by Western blot (*n* = 4). **(E,F)** Isolated adipose tissue from normal mice were cultured in DMEM and treated with Er-Miao-Fang (EMF, 100 μg/mL), TNF-α antibody (1 μg/mL), or IL-1β antibody (0.6 μg/mL) for 30 min before challenged with PA (100 μM) for 24 h. PDE3B expression and cAMP contents in isolated adipose tissue were determined by Western blot (*n* = 4) or ELISA (*n* = 5∼6), respectively. The results were expressed as the mean ± SD. ^∗^*p* < 0.05 vs. HFD feeding or PA challenge only treatment, #*p* < 0.05 vs. the indicated treatment.

Adipose dysfunction is associated with inflammation. HFD feeding evoked inflammatory response, whereas Er-Miao-Fang and metformin inhibited inflammation in adipose tissue by attenuating JNK phosphorylation and reducing TNF-α and IL-1β production (**Figures 2A–C**). The protein expression of PDE3B was decreased in adipose tissue of HFD-fed mice. Nevertheless, administration of Er-Miao-Fang and metformin preserved PDE3B induction (**Figure 2D**). To investigate the impact of inflammation on lipolysis, we isolated adipose tissue from normal mice and treated with saturated fatty acid palmitate (PA). PA stimulation inhibited PDE3B expression and increased cAMP accumulation, but these alternations were reversed by Er-Miao-Fang treatment at concentration of 100 μg/ml (**Figures 2E,F**). Neutralizing pro-inflammatory cytokines TNF-α and IL-1β with special antibodies preserved PDE3B protein expression and decreased cAMP accumulation in adipose tissue (**Figures 2E,F**).

### Er-Miao-Fang Improved Pyruvate Tolerance in HFD-fed Mice

**FIGURE 3:**
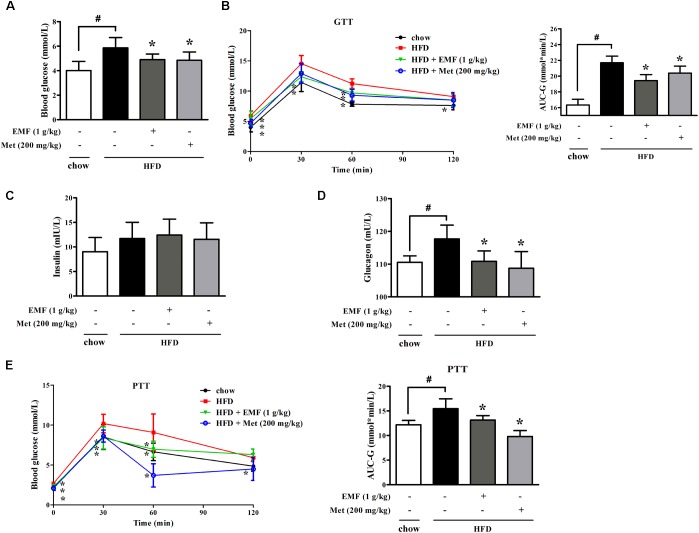
Oral administration of Er-Miao-Fang inhibited endogenous glucose production in HFD-fed mice. Mice were fed with chow diet or HFD for 10 days with oral administration of Er-Miao-Fang extracts (EMF, 1 g/kg) or metformin (Met, 200 mg/kg). **(A)** Fasting blood glucose levels were determined by biochemical kits. **(B)** Blood glucose levels and AUC during the oral glucose tolerance test **(C,D)** Blood insulin and glucagon contents were detected by ELISA. **(E)** Blood glucose levels and AUC during the pyruvate tolerance test. Data were expressed as the mean ± SD (*n* = 10). ^∗^*p* < 0.05 vs. HFD feeding only treatment, #*p* < 0.05 vs. the indicated treatment. GTT, glucose tolerance test; PTT, pyruvate tolerance test.

High-fat-diet feeding increased fasting blood glucose with glucose intolerance in mice. Oral administration of Er-Miao-Fang and metformin reduced fasting blood glucose with improved glucose tolerance in HFD-fed mice (**Figures 3A,B**). Meanwhile, we observed that Er-Miao-Fang decreased the levels of blood glucagon without affecting insulin contents in the blood (**Figures 3C,D**). Pyruvate tolerance test is an indicator of endogenous glucose production since pyruvate load provides the substrate for hepatic glucose production through gluconeogenesis. The changes in glucose levels after a challenge with pyruvate revealed higher glucose levels in HFD-fed mice when compared with chow-fed mice, indicative of impaired pyruvate tolerance (**Figure 3E**). Oral administration of Er-Miao-Fang and metformin reversed pyruvate intolerance in HFD-fed mice (**Figure 3E**). Consistent with inhibitory effect on adipose lipolysis, Er-Miao-Fang downregulated the elevated levels of blood free fatty acids and glycerol without affecting other biochemical parameters in the blood (**Supplementary Figure 1**). These results suggested the inhibitory effect on endogenous glucose production.

### Er-Miao-Fang Blocked Hepatic cAMP and PKA Induction

**FIGURE 4:**
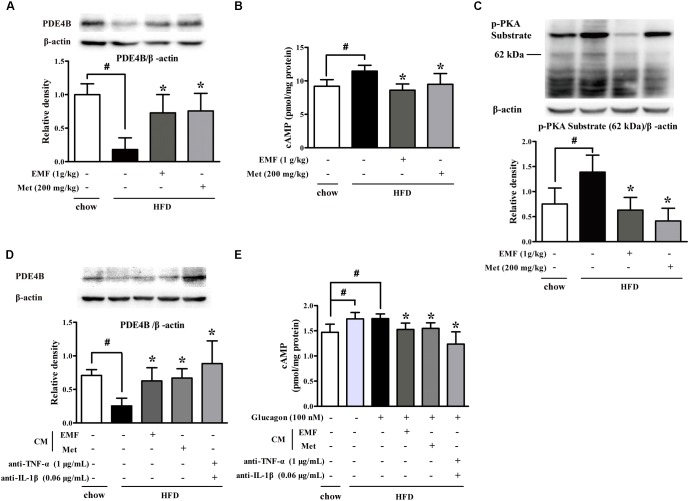
Er-Miao-Fang blocked hepatic cAMP and PKA induction. Mice were fed with chow diet or HFD for 10 days with oral administration of Er-Miao-Fang extracts (EMF, 1 g/kg) or metformin (Met, 200 mg/kg). BNL CL.2 hepatocytes were cultured in fat-derived CM from chow or HFD feeding mice or TNF-α antibody (1 μg/mL) and IL-1β antibody (0.6 μg/mL) treated with or without glucagon (100 nM) for 24 h. **(A)** PDE4B expression in liver tissue were detected by Western blot. The results were showed as the mean ± SD from four independent experiments. **(B)** cAMP in liver tissue were determined by ELISA kits (mean ± SD, *n* = 9∼10). **(C)** Phosphorylation of PKA substrate in liver tissue was detected by Western blot. The results were showed as the mean ± SD from four independent experiments. **(D)** PDE4B expression in hepatocytes were determined by Western blot (*n* = 3) and **(E)** cAMP contents in the present of glucagon (100 nM) were measured by ELISA kits (*n* = 6). The results were expressed as the mean ± SD. ^∗^*p* < 0.05 vs. HFD feeding or HFD-CM or HFD-CM with glucagon treatment, ^#^*p* < 0.05 vs. the indicated treatment. CM, conditioned medium.

Next, we examined hepatic gluconeogenesis in HFD-fed mice, and found that HFD feeding attenuated PDE4B protein expression with cAMP accumulation in the liver (**Figures 4A,B**). Er-Miao-Fang and metformin preserved PDE4B protein expression and then effectively reduced cAMP accumulation by preserving PDE4B induction, resultantly inhibiting PKA activity (**Figures 4A–C**). In view of the contribution of adipose dysfunction to hepatic gluconeogenesis (Perry et al., 2014), we prepared CM by incubation of adipose tissue of chow or HFD feeding mice and then incubated with hepatocytes. BNLCL.2 hepatocytes treated with HFD feeding mice-derived CM decreased PDE4B expression and increased glucagon-mediated cAMP accumulation in hepatocytes, but these effects were attenuated by co-treatment with TNF-α and IL-1β antibodies, suggesting that adipose dysfunction-derived inflammatory mediators impaired hepatic PDE4B to prevent cAMP degradation (**Figures 4D,E**). As expected, Er-Miao-Fang or metformin-derived CM effectively restored PDE4B protein expression and thus reduced cAMP accumulation in response to glucagon (**Figures 4D,E**). These results provided evidence that hepatic PDE4B induction was also impaired by inflammation partly and amelioration of adipose dysfunction might have the potential contribution to attenuate hepatic glucagon signaling.

### Er-Miao-Fang Restrained Hepatic Glucagon Response

**FIGURE 5:**
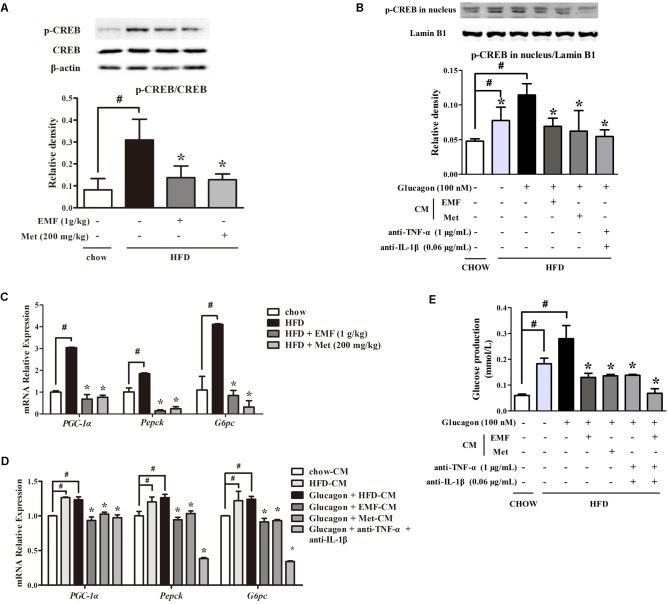
Er-Miao-Fang restrained hepatic glucagon response. Mice were fed with chow diet or HFD for 10 days with oral administration of Er-Miao-Fang (EMF, 1 g/kg) or metformin (Met, 200 mg/kg). BNL CL.2 hepatocytes were cultured in fat-derived CM from chow or HFD feeding mice or TNF-α antibody (1 μg/mL) and IL-1β antibody (0.6 μg/mL) treated with glucagon (100 nM) for 24 h. **(A,B)** CREB protein expression in liver tissue and the phosphorylation of CREB in hepatic nucleus were determined by Western blot (mean ± SD, *n* = 3) **(C,D)** mRNA expressions of *PGC-1α*, *Pepck*, and *G6pc* in liver tissue or hepatocytes were measured by quantitative real time RT-PCR (Q-PCR). Data were mean ± SD of triplicate determinations, which were repeated three times with similar results. **(E)** Hepatic glucose production in response to glucagon in BNL CL.2 hepatocytes treated with fat-derived CM with or without TNF-α antibody (1 μg/mL) and IL-1β antibody (0.6 μg/mL) in the presence or absent of glucagon (100 nM). The results were showed as the mean ± SD (*n* = 6). ^∗^*p* < 0.05 vs. HFD feeding or HFD-CM with glucagon treatment, #*p* < 0.05 vs. the indicated treatment. CM, conditioned medium.

In response to cAMP/PKA signaling, the transcription factor cAMP-response element binding protein (CREB) is activated indicated by increased phosphorylation. Consistently, we observed CREB activation in liver of HFD-fed mice; however, oral administration of Er-Miao-Fang and metformin inhibited CREB activation by dephosphorylation (**Figure 5A**). To confirm the involvement of inflammation in CREB activation and downstream gene regulation, we co-incubated hepatocytes with adipose-derived CM. Glucagon promoted phosphorylated CREB translocation into the nucleus and this action was enhanced by co-treatment with CM derived from HFD-fed mice, but the increased phosphorylated CREB in the nucleus was reduced by neutralizing TNF-α and IL-1β with special antibodies (**Figure 5B**). CREB transcriptionally upregulates gene encoding gluconeogenesis. HFD feeding increased gene expressions of *PGC-1*α*, Pepck*, and *G6pc* in the liver, but the increased gene expressions were inhibited by Er-Miao-Fang and metformin administration (**Figure 5C**). When hepatocytes were exposed adipose-derived CM, Er-Miao-Fang and metformin, or TNF-α and IL-1β antibodies, suppressed gluconeogenetic gene expression (**Figure 5D**). We next showed that when hepatocytes were incubated with adipose-derived CM and antibodies of inflammatory factors at the same time, reduced hepatic glucose production in response to glucagon (**Figure 5E**). Together, these results might suggest that Er-Miao-Fang inhibited hepatic glucagon response in HFD-fed mice through inhibiting adipose inflammation.

## Discussion

Adipose dysfunction is closely related to metabolic diseases. Inflammatory mediators, including pro-inflammatory cytokines and FFAs, increase in the contents of FFAs in the blood circulation, linking adipose dysfunction and systemic insulin resistance in diabetes (Unger, 1995; Boden, 1999; Kim et al., 2001; Frayn, 2002). Herein, our work showed that Er-Miao-Fang might prevent adipose lipolysis by suppression of inflammation in adipose tissue, contributing to reducing excessive hepatic glucose output. This provides new insight into the role of Er-Miao-Fang in regulation of hepatic gluconeogenesis and presents the guiding significance for the regulation of multi-link targets with Traditional Chinese Medicine.

In the chief six components of prepared Er-Miao-Fang extracts, the main ingredients are alkaloids (Feng et al., 2017). Berberine and phellodendrine were reported to have the anti-inflammation activation. Short-term HFD induces adipose inflammation and adipose dysfunction, which is the upstream of crosstalk between adipose and liver. We found that berberine or phellodendrine inhibited the release of fatty acids from adipose tissue, while Er-Miao-Fang extracts prevented lipolysis even well. This suggested the potential advantages of Er-Miao-Fang extracts compared to berberine or phellodendrine in inhibiting lipolysis (**Supplementary Figure 2**), and it might be worth to discuss the superiority of Traditional Chinese Medical prescription in further study.

High-fat-diet feeding causes metabolic disorders. Actually, in our studies, lipolysis was induced by activating cAMP/PKA signaling. HFD-feeding enhanced PKA activation with an increase in cAMP accumulation and AMP reduction in adipocytes. As it has been known, cAMP is synthesized by AC and degraded by PDEs. Adenosine nucleosides are shown to inhibit AC activity (Fain et al., 1972). Er-Miao-Fang reduced cAMP accumulation with elevated levels of AMP in the fat of HFD-feeding mice, indicating that Er-Miao-Fang inhibited the expression of PKA and HSL phosphorylation to decreased lipolysis *via* attenuating cAMP/PKA signaling.

Phosphodiesterases activity was attenuated with cAMP accumulation, which was increased by pro-inflammatory cytokine TNF-α and NF-κB inflammatory signaling (Zhang et al., 2002; Degerman et al., 2011; Ke et al., 2015). So, it is necessary to investigate the relation of cAMP/PKA lipolysis signaling and inflammation in HFD-fed mice. cAMP is an important regulator of immune and inflammatory response, and turnover of intracellular cyclic nucleotides is dependent on the activity of PDEs (Oldenburger et al., 2012). In adipose tissue of obesity, TNF-α is found to impair the activity of PDE3B (Mei et al., 2002). Downregulation of PDE3B contributes to the mechanism whereby TNF-α induces lipolysis and excess release of FFAs (Rahn Landström et al., 2000). In our work, inflammation in adipose tissue of HFD-feeding mice was distinct, with the decrease of PDE3B expression. However, the decrease was removed by adding the TNF-α or IL-1β antibodies, indicating the contribution of inflammation to the activation of cAMP/PKA signaling. Er-Miao-Fang inhibited JNK activation with reduced inflammatory TNF-α and IL-1β release, and effectively preserved PDE3B activity. Also, Er-Miao-Fang showed the same effects with the neutralizing antibodies of inflammation factors, presenting that Er-Miao-Fang prevented lipolysis by inhibiting inflammation.

Obesity is characterized by low-grade inflammation, and chronic or continuous inflammation activates NF-κB signaling in adipose tissue and liver (Arkan et al., 2005; Cai et al., 2005; Donath and Shoelson, 2011). PEDs express not only in adipose, but also in liver (Abdollahi et al., 2003; Ke et al., 2015), and hepatic cAMP degradation is predominantly caused by PDE4B (Miller et al., 2013; Johanns et al., 2016). Recent studies demonstrated that PDE4B expression was associated with enhanced NF-κB activation and transcriptional activity. Indeed, we observed that expression of PDE4B was reduced in liver with adipose dysfunction. So, we next demonstrated that TNF-α and IL-1β antibodies preserved the PDE4B expression in BNL CL.2 hepatocytes, which was decreased by HFD fat-derived CM. Simultaneously, TNF-α and IL-1β antibodies decreased cAMP accumulation in hepatocytes activated by glucagon and HFD fat-derived CM. This data implied that adipose inflammation might link to the PDE4B expression, which induced hepatic cAMP degradation.

Er-Miao-Fang improved pyruvate tolerance in the HFD-fed mice, demonstrating its inhibitory effect on endogenous glucose production. Glucagon increases hepatic glucose output through gluconeogenesis mediated by activating cAMP/PKA signaling, which phosphorylates CREB protein. Phosphorylated CREB, together with different co-activators, translocases into the nucleus and stimulates transcription of gluconeogenic genes (Gonzalez and Montminy, 1989; Ravnskjaer et al., 2007). Metformin, which is the first line therapeutic drug for diabetes to decrease blood glucose, triggers the dissociation of the CREB transcription complex and reduces gluconeogenic enzyme gene expression (He et al., 2009). For this, we observed the effect of Er-Miao-Fang on the glucagon response. Similar to metformin, Er-Miao-Fang decreased the phosphorylation status of CREB, the translocation to nucleus, gluconeogenic enzyme gene expression and glucose production in HFD-feeding mice liver tissue and hepatocytes stimulated by glucagon. Studies documented that overactivation of liver NF-κB-inducing kinase (NIK) enhanced hyperglycemia by increasing CREB stability in obese mice (Sheng et al., 2012). Interestingly, we further demonstrated that TNF-α and IL-1β antibodies lowered that effect, partly indicating the potential pathway that Er-Miao-Fang decreased hepatic cAMP/PKA gluconeogenesis signaling through inhibiting inflammation in HFD-feeding mice. However, this is should be further confirmed by blocking adipose inflammation or adopting adipose-specific PDE3B mutant mice in the future study.

It is noteworthy that inflammation-associated lipolysis occurred in short-term feeding and this could be the potential cause of insulin resistance and hepatic glucose production (Li et al., 2017). Also, our work showed that lipolysis occurred by inhibiting PDE3B and increasing cAMP activity in adipose, and this gives rise to suggesting that the product FFAs may have an effluence to glucose metabolism through other pathways to be proved. Overall, these may partly support the relevance of adipose inflammation and hepatic glucose production. However, the possibility that Er-Miao-Fang ameliorates inflammation in liver could not be excluded. Given that the feature of Traditional Chinese Medical may be of multi-link, it will be worth to pay attention to the explicit pathway that mediating glucose and lipid metabolism in the future study.

**FIGURE 6:**
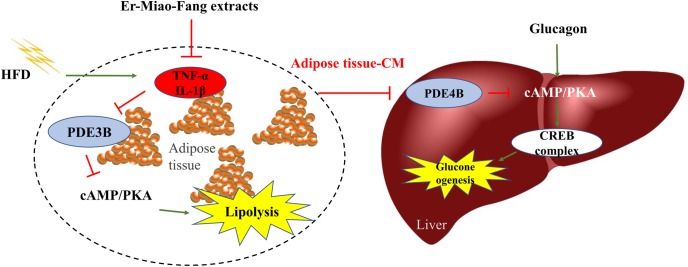
The working pathway for Er-Miao-Fang inhibiting inflammation and blocking hepatic gluconeogenesis. HFD feeding induced inflammation and increased hepatic glucagon response, which leading to endogenous glucose production. Er-Miao-Fang inhibited inflammation and improved adipose dysfunction by preserving PDE3B and decreasing the accumulation of cAMP to prevent lipolysis. By blocking the traffic of fatty acids and inflammatory mediators from adipose tissue to the liver, Er-Miao-Fang inhibited the effect on hepatic gluconeogenesis through preserving PDE4B and reducing cAMP/PKA signaling.

In summary, inflammation in adipose tissue might act as an important mediator to induce lipolysis and hepatic gluconeogenesis. Traditional Chinese Medical prescription Er-Miao-Fang prevented inflammation in adipose tissue and subsequent inhibited hepatic cAMP accumulation in liver contributing to restraint of the hepatic glucagon response (**Figure 6**). This study presents a new view of inhibiting inflammation to ameliorate glucose homeostasis and provides the guiding significance for the regulation of multi-link targets with Traditional Chinese Medicine.

## Author Contributions

NZ, BL, and JX designed the research. WZ performed experiments, analyzed, interpreted data, and drafted the manuscript. XF collected the data and reviewed the manuscript. NZ and JX edited the manuscript. BL contributed to the discussion of the manuscript. All authors approved the final version of the paper.

## Supplementary Material

The Supplementary Material for this article can be found online at: https://www.frontiersin.org/articles/10.3389/fphys.2018.01041/full#supplementary-material

## Conflict of Interest Statement

The authors declare that the research was conducted in the absence of any commercial or financial relationships that could be construed as a potential conflict of interest.

## Abbreviations

cAMP: cyclic adenosine monophosphate
CREB: cAMP-response element binding protein
G6pase: glucose-6-phosphatase
HSL: hormone-sensitive lipase
PA: palmitic acid
PDE3B: phosphodiesterase 3B
PDE4B: phosphodiesterase 4B
PEPCK: phosphoenolpyruvate carboxykinase
PGC-1α: PPARγ coactivator 1α

## References

[B1] Abdollahi M. Chan T. S. Subrahmanyam V. O’Brien P. J. (2003). Effects of phosphodiesterase 3,4,5 inhibitors on hepatocyte cAMP levels, glycogenolysis, gluconeogenesis and susceptibility to a mitochondrial toxin. *Mol. Cell. Biochem.* 252 205–211. 14577594 10.1023/a:1025568714217

[B2] Arkan M. C. Hevener A. L. Greten F. R. Maeda S. Li Z. W. Long J. M. (2005). IKK-β links inflammation to obesity-induced insulin resistance. *Nat. Med.* 11 191–198. 10.1038/nm1185 15685170

[B3] Bae S. Jung Y. Choi Y. M. Li S. (2015). Effects of er-miao-san extracts on TNF-alpha-induced MMP-1 expression in human dermal fibroblasts. *Biol. Res.* 48:8. 10.1186/0717-6287-48-8 25761492 PMC4417304

[B4] Boden G. (1999). Free fatty acids, insulin resistance, and type 2 diabetes mellitus. *Proc. Assoc. Am. Physicians* 111 241–248. 10.1046/j.1525-1381.1999.99220.x 10354364

[B5] Brasaemle D. L. (2007). Thematic review series: adipocyte biology. The perilipin family of structural lipid droplet proteins: stabilization of lipid droplets and control of lipolysis. *J. Lipid Res.* 48 2547–2559. 10.1194/jlr.R700014-JLR200 17878492

[B6] Cai D. Yuan M. Frantz D. F. Melendez P. A. Hansen L. Lee J. (2005). Local and systemic insulin resistance resulting from hepatic activation of IKK-β and NF-κB. *Nat. Med.* 11 183–190. 10.1038/nm1166 15685173 PMC1440292

[B7] Chen G. Li K. Fung C. Liu C. Wong H. Leung P. (2014). Er-Miao-San, a traditional herbal formula containing Rhizoma Atractylodis and Cortex Phellodendri inhibits inflammatory mediators in LPS-stimulated RAW264.7 macrophages through inhibition of NF-κB pathway and MAPKs activation. *J. Ethnopharmacol.* 154 711–718. 10.1016/j.jep.2014.04.042 24815219

[B8] Degerman E. Ahmad F. Chung Y. W. Guirguis E. Omar B. Stenson L. (2011). From PDE3B to the regulation of energy homeostasis. *Curr. Opin. Pharmacol.* 11 676–682. 10.1016/j.coph.2011.09.015 22001403 PMC3225700

[B9] Donath M. Y. Shoelson S. E. (2011). Type 2 diabetes as an inflammatory disease. *Nat. Rev. Immunol.* 11 98–107. 10.1038/nri2925 21233852

[B10] Fain J. N. Pointer R. H. Ward W. F. (1972). Effects of adenosine nucleosides on adenylate cyclase, phosphodiesterase, cyclic adenosine monophosphate accumulation, and lipolysis in fat cells. *J. Biol. Chem.* 247 6866–6872. 4343159

[B11] Feng X. Zhao W. Hou T. Zhang N. (2017). Simultaneous determination of 14 compounds in Er Miao San extracts by UPLC-MS/MS. *Zhongguo Shi Yan Fang Ji Xue Za Zhi* 23 116–122.

[B12] Francis S. H. Blount M. A. Corbin J. D. (2011). Mammalian cyclic nucleotide phosphodiesterases: molecular mechanisms and physiological functions. *Physiol. Rev.* 91 651–690. 10.1152/physrev.00030.2010 21527734

[B13] Frayn K. N. (2002). Adipose tissue as a buffer for daily lipid flux. *Diabetologia* 45 1201–1210. 10.1007/s00125-002-0873-y 12242452

[B14] Gauthier M. S. Miyoshi H. Souza S. C. Cacicedo J. M. Saha A. K. Greenberg A. S. (2008). AMP-activated protein kinase is activated as a consequence of lipolysis in the adipocyte: potential mechanism and physiological relevance. *J. Biol. Chem.* 283 16514–16524. 10.1074/jbc.M708177200 18390901 PMC2423258

[B15] Gonzalez G. A. Montminy M. R. (1989). Cyclic AMP stimulates somatostatin gene transcription by phosphorylation of CREB at serine 133. *Cell* 59 675–680. 10.1016/0092-8674(89)90013-5 2573431

[B16] Greenberg A. S. Coleman R. A. Kraemer F. B. McManaman J. L. Obin M. S. Puri V. (2011). The role of lipid droplets in metabolic disease in rodents and humans. *J. Clin. Invest.* 121 2102–2110. 10.1172/JCI46069 21633178 PMC3104768

[B17] Greenberg A. S. Shen W. J. Muliro K. Patel S. Souza S. C. Roth R. A. (2001). Stimulation of lipolysis and hormone-sensitive lipase via the extracellular signal-regulated kinase pathway. *J. Biol. Chem.* 276 45456–45461. 10.1074/jbc.M104436200 11581251

[B18] He L. Sabet A. Djedjos S. Miller R. Sun X. Hussain M. A. (2009). Metformin and insulin suppress hepatic gluconeogenesis through phosphorylation of CREB binding protein. *Cell* 137 635–646. 10.1016/j.cell.2009.03.016 19450513 PMC2775562

[B19] Johanns M. Lai Y. C. Hsu M. F. Jacobs R. Vertommen D. Van Sande J. (2016). AMPK antagonizes hepatic glucagon-stimulated cyclic AMP signalling via phosphorylation-induced activation of cyclic nucleotide phosphodiesterase 4B. *Nat. Commun.* 7:10856. 10.1038/ncomms10856 26952277 PMC4786776

[B20] Ke B. Zhao Z. Ye X. Gao Z. Manganiello V. Wu B. (2015). Inactivation of NF-κB p65 (RelA) in liver improves insulin sensitivity and inhibits cAMP/PKA pathway. *Diabetes Metab. Res. Rev.* 64 3355–3362. 10.2337/db15-0242 26038580 PMC4587638

[B21] Kim J. K. Fillmore J. J. Chen Y. Yu C. Moore I. K. Pypaert M. (2001). Tissue-specific overexpression of lipoprotein lipase causes tissue-specific insulin resistance. *Proc. Natl. Acad. Sci. U.S.A.* 98 7522–7527. 10.1073/pnas.121164498 11390966 PMC34701

[B22] Kowalski G. M. De Souza D. P. Burch M. L. Hamley S. Kloehn J. Selathurai A. (2015). Application of dynamic metabolomics to examine in vivo skeletal muscle glucose metabolism in the chronically high-fat fed mouse. *Biochem. Biophys. Res. Commun.* 462 27–32. 10.1016/j.bbrc.2015.04.096 25930998

[B23] Lafontan M. Girard J. (2008). Impact of visceral adipose tissue on liver metabolism. Part I: heterogeneity of adipose tissue and functional properties of visceral adipose tissue. *Diabetes Metab.* 34 317–327. 10.1016/j.diabet.2008.04.001 18550411

[B24] Leahy P. Crawford D. R. Grossman G. Gronostajski R. M. Hanson R. W. (1999). CREB binding protein coordinates the function of multiple transcription factors including nuclear factor I to regulate phosphoenolpyruvate carboxykinase (GTP) gene transcription. *J. Biol. Chem.* 274 8813–8822. 10.1074/jbc.274.13.8813 10085123

[B25] Lee Y. S. Kim W. S. Kim K. H. Yoon M. J. Cho H. J. Shen Y. (2006). Berberine, a natural plant product, activates AMP-activated protein kinase with beneficial metabolic effects in diabetic and insulin-resistant states. *Diabetes Metab. Res. Rev.* 55 2256–2264. 10.2337/db06-0006 16873688

[B26] Li L. Huang T. Tian C. Xiao Y. Kou S. Zhou X. (2016). The defensive effect of phellodendrine against AAPH-induced oxidative stress through regulating the AKT/NF-κB pathway in zebrafish embryos. *Life Sci.* 157 97–106. 10.1016/j.lfs.2016.05.032 27234894

[B27] Li L. Z. Zhang T. Yang L. Zhang L. Wang L. Liu B. (2017). Inhibition of lipolysis by ilexgenin A via AMPK activation contributes to the prevention of hepatic insulin resistance. *Eur. J. Pharmacol.* 813 84–93. 10.1016/j.ejphar.2017.07.038 28739087

[B28] Mei J. Holst L. S. Landström T. R. Holm C. Brindley D. Manganiello V. (2002). C(2)-ceramide influences the expression and insulin-mediated regulation of cyclic nucleotide phosphodiesterase 3B and lipolysis in 3T3-L1 adipocytes. *Diabetes Metab. Res. Rev.* 51 631–637. 10.2337/diabetes.51.3.631 11872660

[B29] Miller R. A. Chu Q. Xie J. Foretz M. Viollet B. Birnbaum M. J. (2013). Biguanides suppress hepatic glucagon signalling by decreasing production of cyclic AMP. *Nature* 494 256–260. 10.1038/nature11808 23292513 PMC3573218

[B30] Miyoshi H. Souza S. C. Zhang H. H. Strissel K. J. Christoffolete M. A. Kovsan J. (2006). Perilipin promotes hormone-sensitive lipase-mediated adipocyte lipolysis via phosphorylation-dependent and -independent mechanisms. *J. Biol. Chem.* 281 15837–15844. 10.1074/jbc.M601097200 16595669

[B31] Oldenburger A. Roscioni S. S. Jansen E. Menzen M. H. Halayko A. J. Timens W. (2012). Anti-inflammatory role of the cAMP effectors Epac and PKA: implications in chronic obstructive pulmonary disease. *PLoS One* 7:e31574. 10.1371/journal.pone.0031574 22363678 PMC3283666

[B32] Perry R. J. Samuel V. T. Petersen K. F. Shulman G. I. (2014). The role of hepatic lipids in hepatic insulin resistance and type 2 diabetes. *Nature* 510 84–91. 10.1038/nature13478 24899308 PMC4489847

[B33] Rahn Landström T. Mei J. Karlsson M. Manganiello V. Degerman E. (2000). Down-regulation of cyclic-nucleotide phosphodiesterase 3B in 3T3-L1 adipocytes induced by tumour necrosis factor alpha and cAMP. *Biochem. J.* 346(Pt 2) 337–343. 10.1042/bj3460337 10677351 PMC1220858

[B34] Ravnskjaer K. Kester H. Liu Y. Zhang X. Lee D. Yates J. R. III (2007). Cooperative interactions between CBP and TORC2 confer selectivity to CREB target gene expression. *EMBO J.* 26 2880–2889. 10.1038/sj.emboj.7601715 17476304 PMC1894761

[B35] Schweiger M. Eichmann T. O. Taschler U. Zimmermann R. Zechner R. Lass A. (2014). Measurement of lipolysis. *Methods Enzymol.* 538 171–193. 10.1016/B978-0-12-800280-3.00010-4 24529439 PMC4018506

[B36] Sheng L. Zhou Y. Chen Z. Ren D. Cho K. W. Jiang L. (2012). NF-κB-inducing kinase (NIK) promotes hyperglycaemia and glucose intolerance in obesity by augmenting glucagon action. *Nat. Med.* 18 943–949. 10.1038/nm.2756 22581287 PMC3766969

[B37] Streeper R. S. Hornbuckle L. A. Svitek C. A. Goldman J. K. Oeser J. K. O’Brien R. M. (2001). Protein kinase A phosphorylates hepatocyte nuclear factor-6 and stimulates glucose-6-phosphatase catalytic subunit gene transcription. *J. Biol. Chem.* 276 19111–19118. 10.1074/jbc.M101442200 11279202

[B38] Unger R. H. (1995). Lipotoxicity in the pathogenesis of obesity-dependent NIDDM. Genetic and clinical implications. *Diabetes* 44 863–870. 10.2337/diab.44.8.863 7621989

[B39] Unger R. H. Cherrington A. D. (2012). Glucagonocentric restructuring of diabetes: a pathophysiologic and therapeutic makeover. *J. Clin. Invest.* 122 4–12. 10.1172/JCI60016 22214853 PMC3248306

[B40] Wang L. Zhang B. Huang F. Liu B. Xie Y. (2016). Curcumin inhibits lipolysis via suppression of ER stress in adipose tissue and prevents hepatic insulin resistance. *J. Lipid Res.* 57 1243–1255. 10.1194/jlr.M067397 27220352 PMC4918853

[B41] Wei S. Zhang M. Yu Y. Lan X. Yao F. Yan X. (2016). Berberine attenuates development of the hepatic gluconeogenesis and lipid metabolism disorder in type 2 diabetic mice and in palmitate-incubated HepG2 cells through suppression of the HNF-4α miR122 pathway. *PLoS One* 11:e0152097. 10.1371/journal.pone.0152097 27011261 PMC4806913

[B42] Xiao N. Lou M. Lu Y. Yang L. Liu Q. Liu B. (2017). Ginsenoside Rg5 attenuates hepatic glucagon response via suppression of succinate-associated HIF-1α induction in HFD-fed mice. *Diabetologia* 60 1084–1093. 10.1007/s00125-017-4238-y 28280902

[B43] Yang H. Yang L. (2016). Targeting cAMP/PKA pathway for glycemic control and type 2 diabetes therapy. *J. Mol. Endocrinol.* 57 R93–R108. 10.1530/JME-15-0316 27194812

[B44] Zhang H. H. Halbleib M. Ahmad F. Manganiello V. C. Greenberg A. S. (2002). Tumor necrosis factor-α stimulates lipolysis in differentiated human adipocytes through activation of extracellular signal-related kinase and elevation of intracellular cAMP. *Diabetes* 51 2929–2935. 10.1152/ajpendo.00228.2012 12351429

[B45] Zhao W. Li A. Feng X. Hou T. Liu K. Liu B. (2016). Metformin and resveratrol ameliorate muscle insulin resistance through preventing lipolysis and inflammation in hypoxic adipose tissue. *Cell. Signal.* 28 1401–1411. 10.1016/j.cellsig.2016.06.018 27343375

[B46] Zhao W. Wang M. Shao L. Liao M. Liu K. Huang F. (2014). The total phenolic fraction of Anemarrhena asphodeloides inhibits inflammation and reduces insulin resistance in adipocytes via regulation of AMP-kinase activity. *Planta Med.* 80 146–152. 10.1055/s-0033-1360197 24431016

