# Supplementary material for: Er-Miao-Fang Extracts Inhibits Adipose Lipolysis and Reduces Hepatic Gluconeogenesis via Suppression of Inflammation
Source: Front Physiol. 2018 Aug 14;9:1041. doi: 10.3389/fphys.2018.01041 (PMC6102449; doi:10.3389/fphys.2018.01041)
Supplement: Supplementary file 1 [file Data_Sheet_1.ZIP › supplementary material/supproting information.docx]

**Methods**

**Effect of Er-Miao-Fang treatment in HFD-fed mice**

Mice were fed a regular chow diet or HFD for 10 days with oral administration of saline, Er-Miao-Fang extracts (1 g/kg) or metformin (200 mg/kg) respectively every day. Body weight and food intake were recorded. chow-fed and HFD-fed mice were fasted for 8 h before collecting blood from the orbital sinus. Free fatty acids (FFAs, Jiancheng, Nanjing, China), and Glycerol (Jiancheng, Nanjing, China) contents in blood were assayed according to the manufacturer’s instructions.

**Figure legends**

**Sup Figure 1 Effect of Er-Miao-Fang treatment in HFD-fed mice.** Mice were fed with chow diet or HFD for 10 days with oral administration of Er-Miao-Fang extracts (EMF, 1 g/kg) or metformin (Met, 200 mg/kg). (**A**) Body weights of chow-fed and HFD-fed mice every day (mean ± SD, n=10). (**B**) Food intake of per mouse every day. Data were expressed as the mean ± SD (n=3). (**C, D**) Serum were collected after mice were sacrificed. Free fatty acids (FFAs) and glycerol were measured by commercial assay kits (mean ± SD, n=7~9). **p*<0.05 *vs* HFD feeding only treatment, ^#^*p*<0.05 *vs* the indicated treatment.

**Sup Figure 2 Er-Miao-Fang extracts and its main elements inhibited the release of FFAs from adipose tissue.** Isolated adipose tissue from normal mice were cut into small pieces, cultured in DMEM and treated with Er-Miao-Fang extracts (EMF, 100 μg/mL), berberine (10 μM) or phellodendrine (10 μM) for 30 min before challenged with or without PA (100 μM) for 2 h. After washing, the adipose tissue was incubated in DMEM for another 22 h. The contents of free fatty acids (FFAs) released from adipose tissue were detected. The results were expressed as the mean ±SD (n=6). ^*^p<0.05 *vs* PA only treatment, ^#^p<0.05 *vs* the indicated treatment. BBR, berberine; OB-5, phellodendrine.
