# Supplementary figures and images for: Er-Miao-Fang Extracts Inhibits Adipose Lipolysis and Reduces Hepatic Gluconeogenesis via Suppression of Inflammation
Source: Front Physiol. 2018 Aug 14;9:1041. doi: 10.3389/fphys.2018.01041 (PMC6102449; doi:10.3389/fphys.2018.01041)

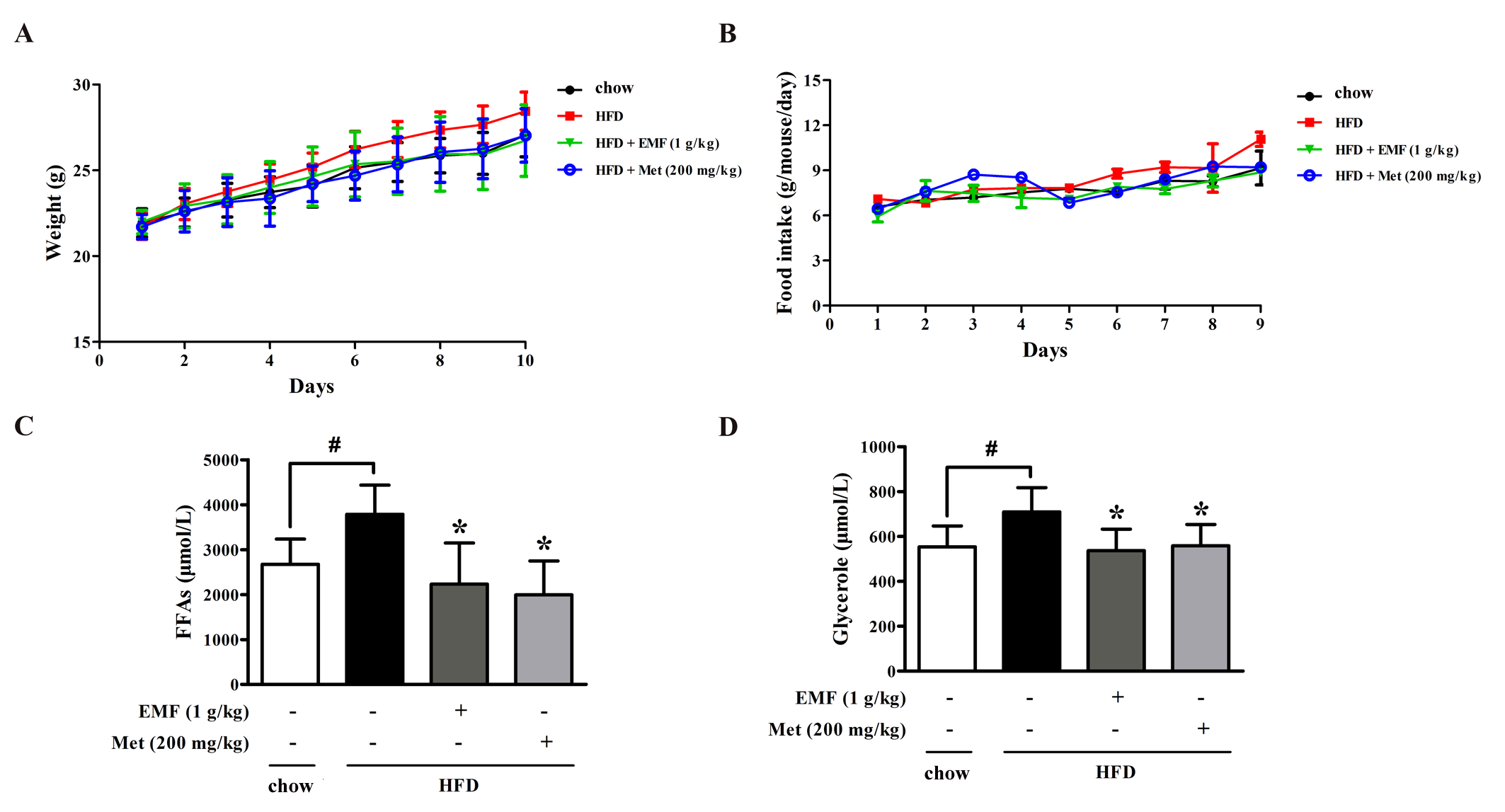

Supplement: Supplementary file 1 [file Data_Sheet_1.ZIP › supplementary material/Sup Figure 1.tif]

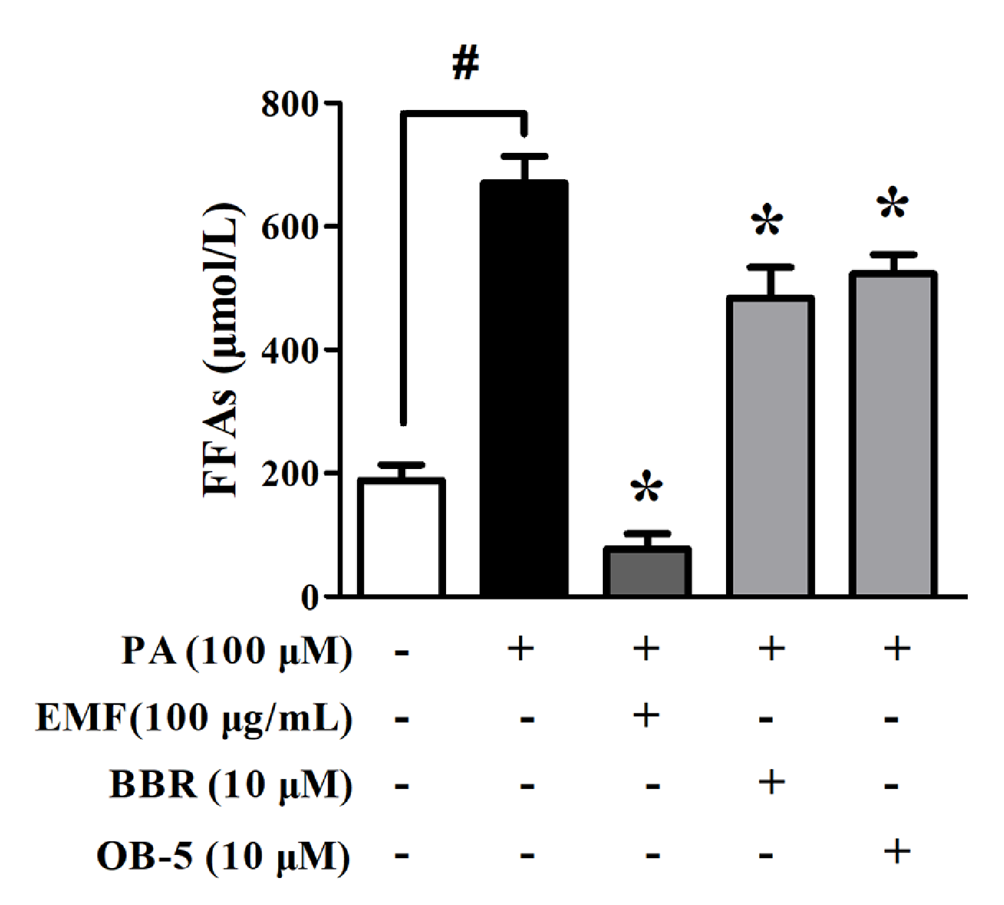

Supplement: Supplementary file 1 [file Data_Sheet_1.ZIP › supplementary material/sup figure 2.tif]
