# Supplementary material for: Er-Miao-Fang Extracts Inhibits Adipose Lipolysis and Reduces Hepatic Gluconeogenesis via Suppression of Inflammation
Source: Front Physiol. 2018 Aug 14;9:1041. doi: 10.3389/fphys.2018.01041 (PMC6102449; doi:10.3389/fphys.2018.01041)

Original images

1. Adipose of HFD – β-actin

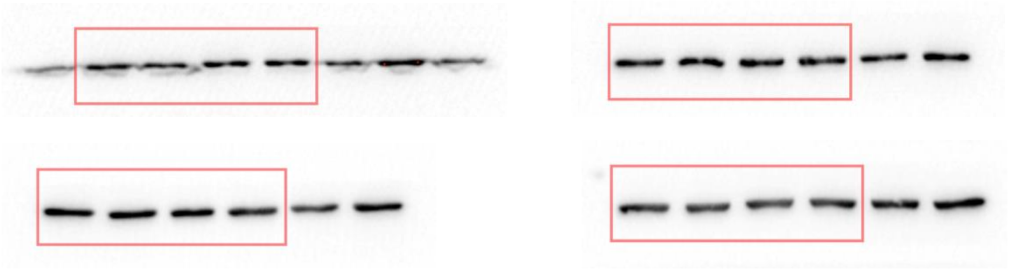

2.adipose tissue of HFD - HSL

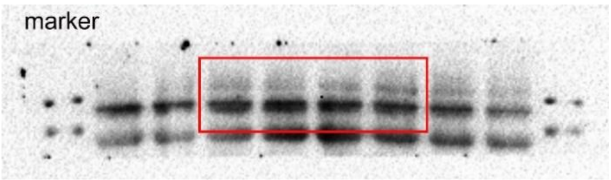

3.adipose tissue of HFD – p-HSL

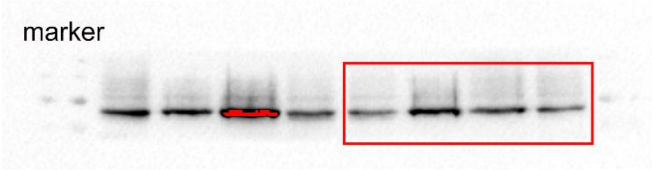

4.adipose tissue of HFD - JNK

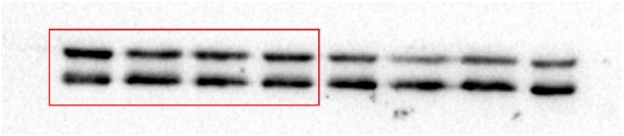

5.adipose tissue of HFD – p-JNK

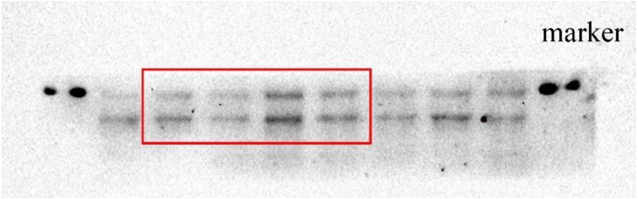

6. adipose tissue of HFD – PDE3B

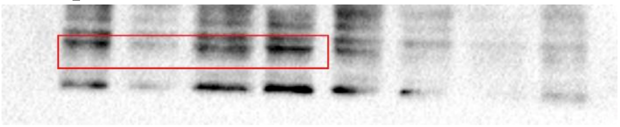

7. adipose tissue of HFD – p-PKA

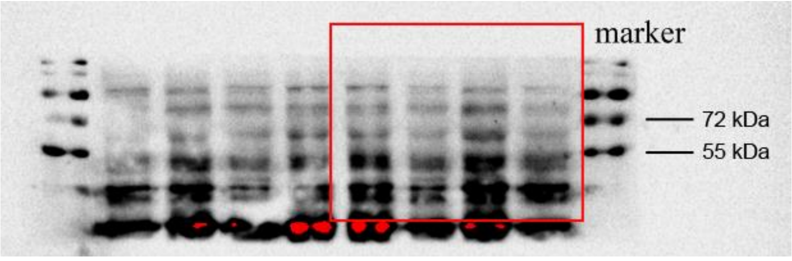

8. Isolated adipose tissue – β-actin

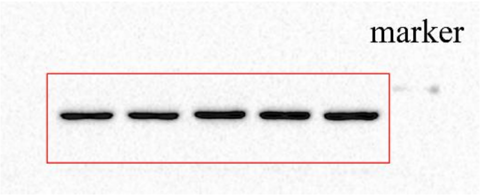

9. Isolated adipose tissue – PDE3B

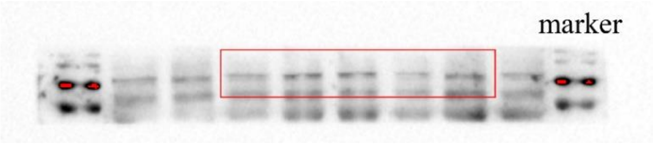

10. BNL CL.2 cells – Lamin B1 in nucleus

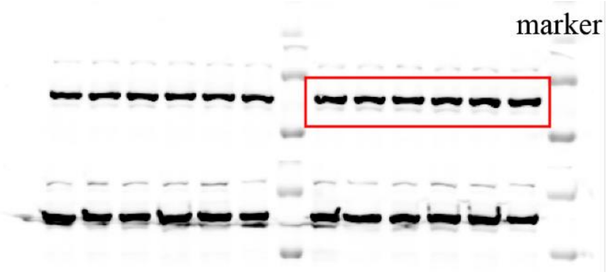

11. BNL CL.2 cells – β-actin

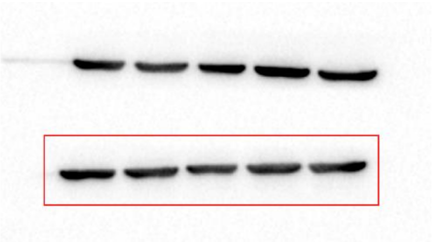

12. BNL CL.2 cells – p-CREB

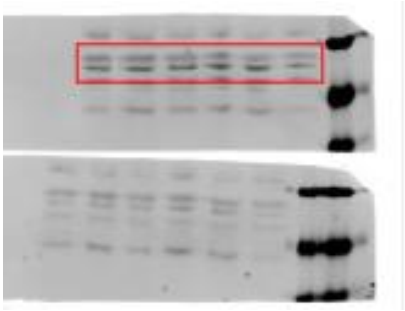

13. BNL CL.2 cells – p-CREB

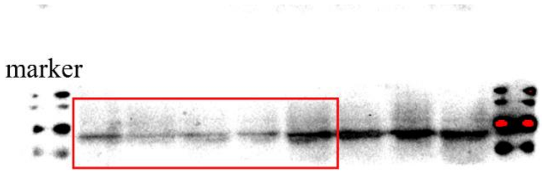

14. liver of HFD - β-actin

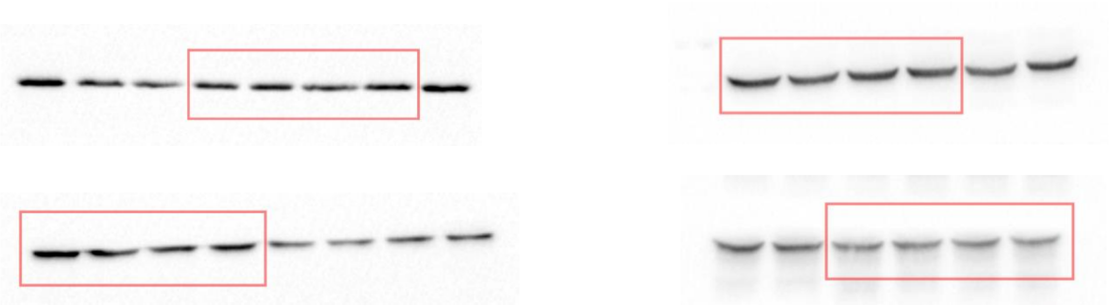

15. liver of HFD - CREB

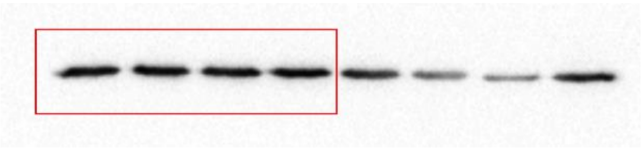

16. liver of HFD -p-CREB

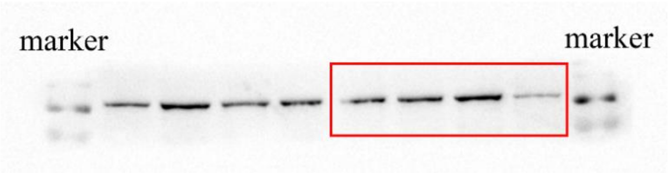

17. liver of HFD -PDE4B

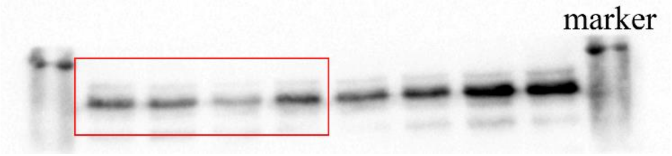

18.

liver of HFD -p-PKA

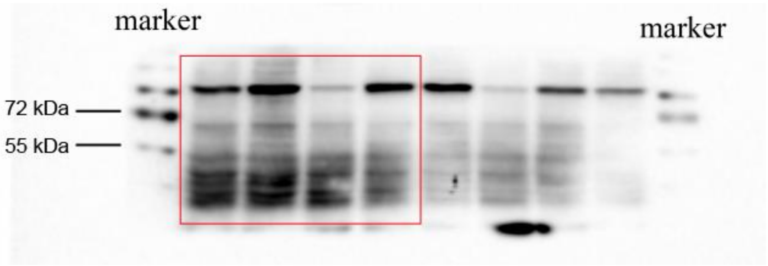

Supplement: Supplementary file 2 [file Image_1.pdf]
